# Characterization, Expression Profiling, and Biochemical Analyses of the *Cinnamoyl-CoA Reductase* Gene Family for Lignin Synthesis in Alfalfa Plants

**DOI:** 10.3390/ijms23147762

**Published:** 2022-07-14

**Authors:** Weiti Cui, Zihan Zhuang, Peihao Jiang, Jincheng Pan, Gan Zhao, Sheng Xu, Wenbiao Shen

**Affiliations:** 1 Laboratory Center of Life Sciences, College of Life Sciences, Nanjing Agricultural University, Nanjing 210095, China; wtcui@njau.edu.cn (W.C.); z15298388017@163.com (Z.Z.); phjiang216@163.com (P.J.); 2018216034@njau.edu.cn (J.P.); 2018216033@njau.edu.cn (G.Z.); 2 Institute of Botany, Jiangsu Province and Chinese Academy of Sciences, Nanjing 210014, China; xusheng@cnbg.net

**Keywords:** *cinnamoyl-CoA reductase*, kinetic analysis, lignin biosynthesis, *Medicago sativa*

## Abstract

*Cinnamoyl-CoA reductase* (*CCR*) is a pivotal enzyme in plant lignin synthesis, which has a role in plant secondary cell wall development and environmental stress defense. Alfalfa is a predominant legume forage with excellent quality, but the lignin content negatively affects fodder digestibility. Currently, there is limited information on CCR characteristics, gene expression, and its role in lignin metabolism in alfalfa. In this study, we identified 30 members in the CCR gene family of *Medicago sativa*. In addition, gene structure, conserved motif, and evolution analysis suggested *MsCCR1–7* presumably functioned as CCR, while the 23 *MsCCR-likes* fell into three categories. The expression patterns of *MsCCRs*/*MsCCR-likes* suggested their role in plant development, response to environmental stresses, and phytohormone treatment. These results were consistent with the *cis*-elements in their promoters. Histochemical staining showed that lignin accumulation gradually deepened with the development, which was consistent with gene expression results. Furthermore, recombinant MsCCR1 and MsCCR-like1 were purified and the kinetic parameters were tested under four substrates. In addition, three-dimensional structure models of MsCCR1 and MsCCR-like1 proteins showed the difference in the substrate-binding motif H212(X)2K215R263. These results will be useful for further application for legume forage quality modification and biofuels industry engineering in the future.

## 1. Introduction

Alfalfa (*Medicago sativa* L.) is a worldwide-cultivated legume forage, with high nutrition and good palatability [1]. Digestibility is part of the key indicators of forage quality, which has a significant influence on animal performance. Generally, forage digestibility is affected by various cellular components, including lignin content [2]. Genetically reducing lignin content in forage legumes can improve digestibility and animal performance, as well as commercialization for forage quality improvement [3]. For example, the *caffeic acid O-methyltransferase* downregulated transgenic tall fescue plants showed reduced lignin content and significantly increased digestibility [4]. When fed adult horses with reduced lignin alfalfa hay, an improvement in the dry matter digestibility was found, with no change in forage consumption, fecal particle size, or digesta retention time [5]. By using transgenic alfalfa lines with downregulated cytochrome P450 enzymes in lignin pathway, it showed a strong negative relationship between lignin content and rumen digestibility but not between lignin composition and digestibility [6]. However, it is difficult to draw a conclusion on whether the changes in lignin monomer could benefit biomass digestibility because there is a lack of unified research background in the previous studies [7]. In addition, lignin also has a negative impact on the conversion of lignocellulose biomass into cellulosic ethanol in the biofuels production industry [8,9,10]. Interestingly, alfalfa is considered a potential feedstock for biofuels due to its being valuable in lignocellulosic biomass [11]. Thus, lignin content modification is an important strategy for both forage and biofuel engineering, with no effect on the plant yield [12].

Lignin is a group of polyphenolic polymers that are deposited predominantly in the thickened secondary cell walls [13]. The evolution of lignin was accompanied by the emergence of the vascular land plants and exhibited both distinct subcellular localization and monomeric composition in specific cell types [14]. Plants with loss-of-function of genes in lignin biosynthesis, such as *cinnamoyl-CoA reductase* (*CCR*), generally showed collapsed xylem and/or dwarf phenotype [15]. Moreover, lignification is one of the responses of plant cells to various environmental stresses. There is a close relationship between lignin accumulation and environmental stresses in many plant species. For example, in different Iranian cultivars of basil, water deficit stress leads to increasing expression of genes in lignin synthesis [16]. Under copper (Cu^2+^) stress condition, *Panax ginseng* suspension cultures showed an increased accumulation of phenolics and lignin, which reflects the protective response to Cu^2+^-induced cell damage [17]. During the plant-environment interaction, the functional integrity of the plant cell needs the maintenance of cell wall integrity. Stresses-induced cell wall damage could initiate the secondary reactive oxygen species (ROS) burst and jasmonic acid (JA) accumulation, followed by a negative feedback loop that represses each other’s production and subsequent lignin accumulation [18]. In addition, lignin content and the expression of lignin-metabolism-related genes were found to play critical roles in lodging resistance of common buckwheat and barley [19,20]. Interestingly, genetically modified plants with decreased lignin biosynthesis can indirectly influence the synthesis of other secondary metabolites and the expression of stress-related genes [21].

Lignin is synthesized in the spaces between the cell wall polysaccharides by the oxidative coupling of three monolignols, coniferyl alcohol, sinapyl alcohol, and *p*-coumaryl alcohol, and then there is incorporation into lignin to form guaiacyl (G), syringyl (S), and *p*-hydroxyphenyl (H) subunits, respectively [14,22]. In plants, lignin monomers are synthesized from phenylalanine in the cytoplasm by two of a total of three branches. The first branch, which is called the general phenylpropanoid pathway, is from phenylalanine to *p*-coumaroyl-CoA and subsequent feruloyl-CoA. This pathway is shared with some important secondary metabolites, such as flavonoids and coumarins [23,24]. The second branch is called monolignol pathway, in which hydroxycinnamoyl-CoA esters are reduced by CCR (EC 1.2.1.44) and cinnamyl alcohol dehydrogenase (CAD; EC 1.1.1.195) to generate monolignols [25]. CCR catalyzes the *p*-coumaryl-CoA, caffeoyl-CoA, and feruloyl-CoA to form *p*-coumaraldehyde, caffeyl aldehyde, and coniferaldehyde, respectively, and is further converted to the corresponding monolignols, coumaryl alcohol, caffeoyl alcohol, and feruloyl alcohol, with the CAD catalyst [14]. Common to all three branches of the lignin synthesis pathway, genetic changes of *CCR* and *CAD* genes tend to exhibit overall reduced lignin content but not one or two monomer(s) [7,26,27]. Therefore, CCR is a pivotal enzyme involved in monolignols biosynthesis in plants.

The *CCR* gene family exhibits diversity in different plant species. Two *CCR* genes and five *CCR-like* genes were found in *Arabidopsis thaliana*. *AtCCR1* plays a role in lignification during the plant developmental stage, and *AtCCR2* is functionally expressed in stress and pathogen response [28,29]. The mutant of *AtCCR1* exhibits decreased lignin content, while triple mutant with the loss-function of *CAD-C*, *CAD-D*, and *CCR1* contains 50% of wild-type lignin content and shows a severe dwarf phenotype and male sterility [30]. Reintroduced *CCR1* expression specifically in the protoxylem and metaxylem vessel cells in the mutant of *AtCCR1* can effectively overcome the vascular collapse and stem biomass yield decrease, regardless of the similar cell wall composition and metabolome compared with *AtCCR1* mutation plants [31]. In switchgrass, PvCCR1 and PvCCR2 were found to possess CCR activity, whereas *PvCCR1* might function in lignin biosynthesis during development stage and *PvCCR2* might be involved in stress defense [32]. In *Populus trichocarpa*, Shi et al. [33] identified 11 CCRs, and PtrCCR2 showed high expression in differentiating xylem and significantly less in other tissues. In model legume *Medicago truncatula*, Zhou et al. [34] have identified two CCR genes, in which *MtCCR1* had a critical effect on plant growth, while *MtCCR2* exhibited moderate reduced lignin content and little influence on growth phenotype. In rice, the *OsCCR* knockdown plants showed a decrease in lignin deposition in root and anther. Interestingly, a component of an SCF E3 ligase OsFBK1 mediated OsCCR degradation, suggesting the role of OsFBK1 and OsCCR in the development of rice anthers and roots [35]. However, there is still little knowledge of the characteristics of the CCR family genes that respond to lignin biosynthesis in widely cultivated forage *M. sativa*.

Cultivated alfalfa is an autotetraploid (2n = 4x = 32), so it generates a lot of trouble in the identification of its genes and molecular genetic research. Recently, alfalfa (*M. sativa* L.) genome sequencing has been completed, in which it was reported with 3010 Mb of genome size [36]. This provides a great opportunity for the genome-wide analysis of the CCR gene family. In this study, we searched the alfalfa genome database to identify CCR family members and further analyzed the gene structures, evolutionary relationships, and gene expression patterns. Finally, we cloned and further recombinantly expressed MsCCR1 and MsCCR-like1, followed by the kinetic parameters and three-dimensional structure analysis, to explore the MsCCRs protein structure and possible function in lignin biosynthesis.

## 2. Results

### 2.1. Identification and Analysis of Cinnamoyl-CoA Reductase (CCR) Genes in M. sativa Genome

In total, 30 putative *CCR* gene sequences were identified from *M. sativa* genome database by domain confirmation and homology research with the signature motif KNWYCYGK. Among them, 7 *CCR* genes were identified with full-length CCR motif sequences, and 23 sequences with partial CCR motifs were characterized as *CCR-like* genes (Table S1). Of the 23 *CCR-like* genes, 7 *CCR-like* genes (namely *MsCCR-like1–7*) are homologous to *M. truncatula CCR-like* genes *SNL6-1/2*, and the other 16 *CCR-like* genes (namely *MsCCR-like8–23*) are homologous to *MtCCR1-3/4*. The molecular weight of 30 predicted MsCCR/MsCCR-like proteins varied from 32.35 to 39.85 kDa, with isoelectric points (pI) in the range of 5.00–7.48.

### 2.2. Gene Structure, Evolution, and Conserved Motif Analysis of MsCCRs/MsCCR-like Genes

**Figure 1:**
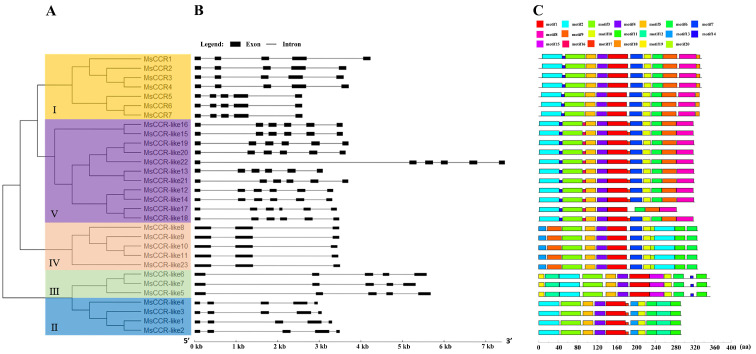
The gene structures and conserved motifs of MsCCR and MsCCR-likes based on evolutionary relationships. Phylogenetic relationships of *MsCCR* and *MsCCR-like* genes (**A**). The exon-intron structure of *MsCCR* and *MsCCR-like* genes (**B**). Distribution of putative conserved motifs in MsCCR and MsCCR-like proteins (**C**).

To find out evolutionary relationships among members of the CCRs from alfalfa, a neighbor-joining (N-J) tree was constructed based on the nucleotide sequences (Figure 1A). The 30 *MsCCRs/MsCCR-likes* sequences were divided into 5 subfamilies, and subfamily I contained all 7 *MsCCR* genes, while *MsCCR-like* genes were classified in subfamilies II–V (Figure 1A). Furthermore, the intron/exon structure of *MsCCRs/MsCCR-like* genes was shown (Figure 1B). It can be clearly seen that genes in the same subfamily have similar gene structures. In turn, there are distinct differences in gene structures among the different subfamilies, with the different numbers of introns from 2–5. For example, subfamily IV has 2 introns, subfamily V has 5 introns, and most of the *CCR* genes (subfamilies I, II, and III) contain 4 introns. By using the MEME online software, 20 conserved motifs in the MsCCRs were captured, and further analyses were carried out (Figure 1C). Furthermore, the sequence of each motif was analyzed (Table S2). All of the MsCCR/MsCCR-like proteins contain motifs 1–6. Proteins in groups I, IV, and V contain motifs 7 and 9, while proteins in groups I and V contain motif 8. Furthermore, proteins in groups I and V mainly lack the motifs 11–13, 15, 19, and 20. In addition, motifs 18, 15, 20, and 16 are unique to proteins in groups I, III, IV, and V, respectively.

**Figure 2:**
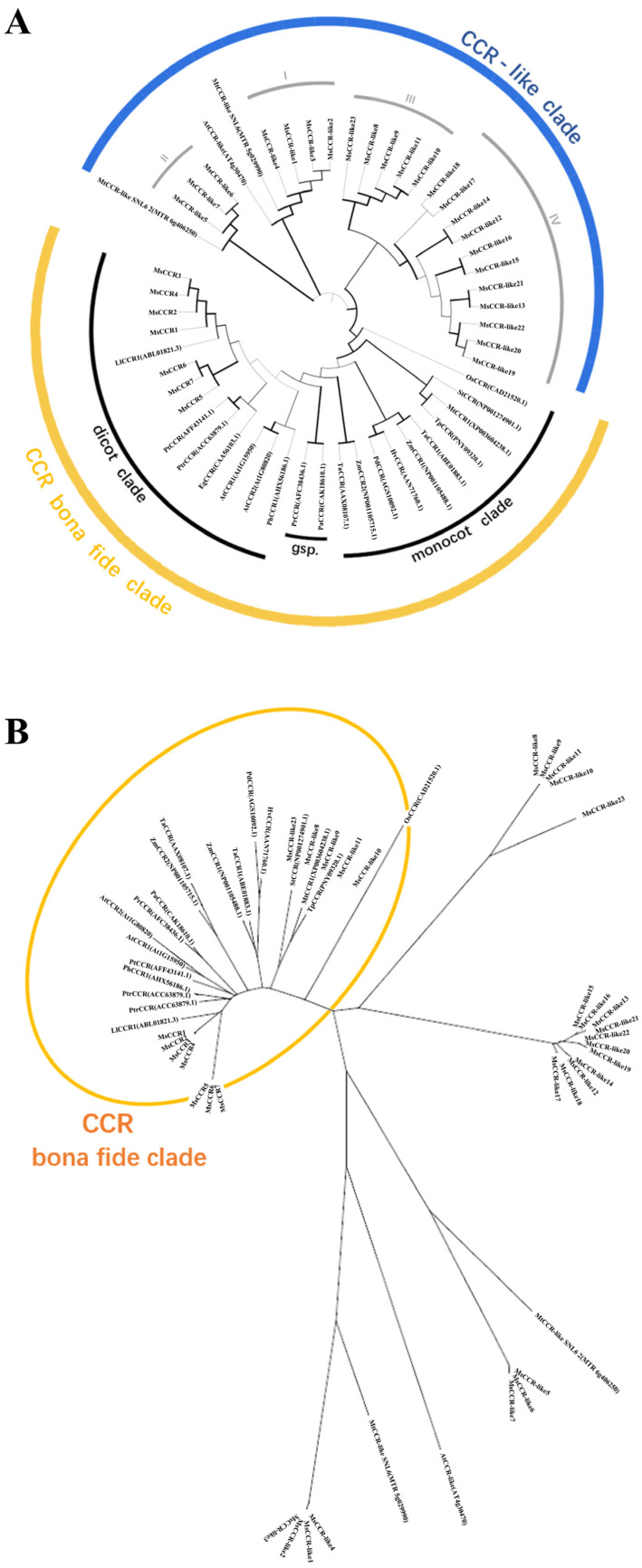
The phylogenetic analysis of CCR proteins across various plants. Traditional phylogenetic tree (**A**) and radiation tree (**B**) of 30 CCR protein sequences from *M. sativa* and 22 CCR protein sequences from other species. Protein sequences were constructed using MRGA-X software based on the neighbor-joining (NJ) method. The species name and the accession number of sequences used to construct the phylogenetic tree are listed in Table S3.

We further constructed two phylogenetic trees with CCRs in alfalfa and other plants to clarify their relationships and predict the possible biological functions (Figure 2). Three CCR-like genes and 19 CCR genes from different plants, including *M. truncatula*, *Arabidopsis thaliana*, *Oryza sativa*, *Zea mays*, *Triticum aestivum*, *Solanum tuberosum*, *Trifolium pretense*, *Leucaena leucocephala*, *Eucalyptus gunnii*, *Pinus radiate*, *Picea abies*, and *Populus trichocarpa*, were selected from NCBI [37] (Table S3). All *CCRs/CCR-like* proteins fell into two groups: the CCR bona fide clade and the CCR-like clade. For example, all the 23 MsCCR-like proteins and one AtCCR-like protein as well as two MtCCR-like SNLs were clustered into the CCR-like clade, and the others were parceled into the CCR bona fide clade. The CCR bona fide clade was further classified into three subfamilies: dicot clade, monocot clade, and gymnosperm clades, and 7 MsCCRs were clustered together with LlCCR1, EgCCR, AtCCR1/2, PtCCR, PtrCCR, and PbCCR1 (Figure 2A). Furthermore, many identified CCRs with the function related to lignin biosynthesis exhibit a close relationship to MsCCRs (in Group I) in the radiation tree (Figure 2B). These results indicated possible functional diversity between CCR and CCR-like proteins.

The chromosome distribution of *CCRs* in *M. sativa* and *M. truncatula* was further analyzed to study the evolutionary mechanism of the *CCR* gene family. *MsCCR1*, *MsCCR2*, *MsCCR3*, and *MsCCR4* were distributed on *M. sativa* chromosome 2.1, 2.2, 2.3, and 2.4, respectively, which were paired with MtCCR1 on *M. truncatula* chromosome 2. *MsCCR5*, *6*, and *7* were distributed on *M. sativa* chromosome 4.1, 4.2, and 4.3, respectively, which were paired to *MtCCR1-2* on *M. truncatula* chromosome 4 (Table S1, Figure S1A). While, *MsCCR-likes* were distributed on *M. sativa* chromosomes 2.1, 2.2, 2.3, 2.4, 4.1, 4.2, 4.3, 4.4, 8.2, 8.3, 5.1, 5.2, 5.3, 5.4, 6.1, 6.2, and 6.3, which were linked to *MtCCR1-3*, *MtCCR1-4*, *MtCCR-like SNL6*, and *MtCCR-like SNL6-2* on *M. truncatula* chromosome 2, 4, 5, and 6, respectively (Table S1, Figure S1B).

**Figure 3:**
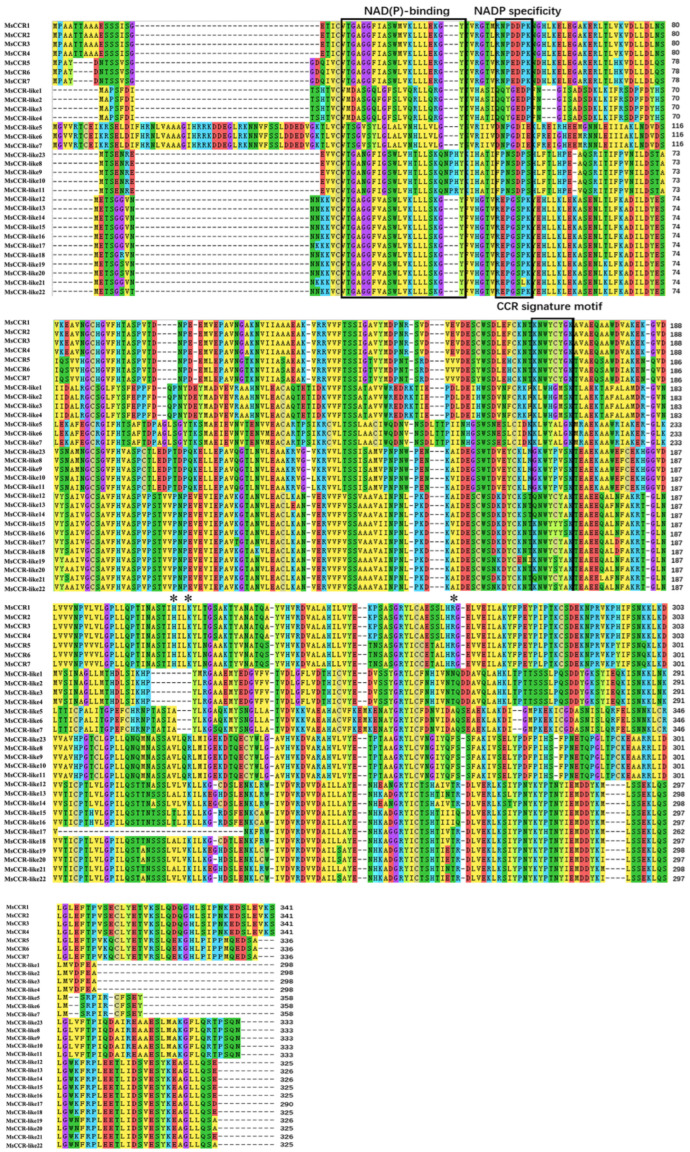
Alignment of MsCCR and MsCCR-like proteins. Conserved motifs are indicated with a black box. “*” indicates the CCR active sites H212, K215, and R263.

Alignments among MsCCR/MsCCR-like proteins revealed that all of them have the NAD(P)-binding motif GXXGXXA/G. MsCCRs and MsCCR-like12–22 also have the NADP^+^ specific motif R(X)_5_K, which was absent in other MsCCR-likes (Figure 3). In addition, only MsCCRs possess the motif H212(X)_2_K215R263 residuce, which was found to be critical for substrate binding in PhCCR1 [38]. Interestingly, these were in agreement with the results in the CCR signature motif KNWYCYGK. The diversity of conserved motifs in MsCCR-like proteins indicates that they might have different activities and biological functions.

### 2.3. The Cis-Acting Regulatory Elements in the Promoter of MsCCRs

**Figure 4:**
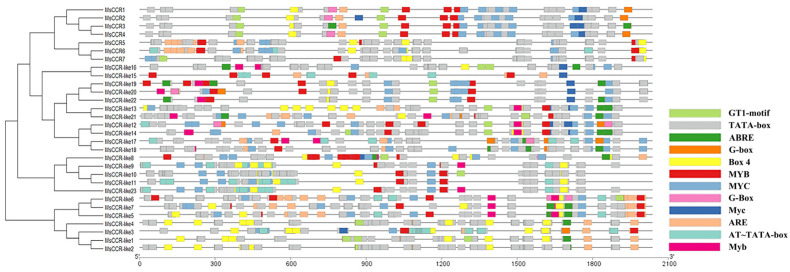
The putative *cis*-elements and transcription factor binding sites in the promoter regions of *MsCCR* and *MsCCR-like* genes. The colored blocks represent different types of *cis*-acting elements and their locations in each *MsCCR*/*MsCCR-like* gene.

The regulation of gene expression in organ cells is a complex process, and transcriptional activation of promoters is a vital event. The promoter of *MsCCR*/*MsCCR-like* genes contained a variety of *cis*-acting elements, including ABRE, ARE, BOX4, G-Box, GT1-motif, MYB, and MYC (Figure 4). For example, the promoters of *MsCCR1–4* have light-related *cis*-acting elements (GT1-motif, Box4, and G-Box), ARE, as well as MYB- and MYC-binding elements, suggesting the expression of *MsCCR1–4* might be involved in light stimulus, anaerobic induction, stress response, and plant development [39]. The elements in promoters of *MsCCR5–7* are comparable, mainly including MYC, Box4, and MYB. Many of the promoters of *MsCCR-likes* possess the elements related to plants responding to stress, such as MYB, MYC, ABRE, ARE, and Box4. These data indicate that *MsCCRs/MsCCR-likes* might participate in plant response to numerous stresses or development-related phytohormone.

### 2.4. The Expression Patterns of MsCCR/MsCCR-like Genes and Lignin Content Analysis during Different Growth Stage

**Figure 5:**
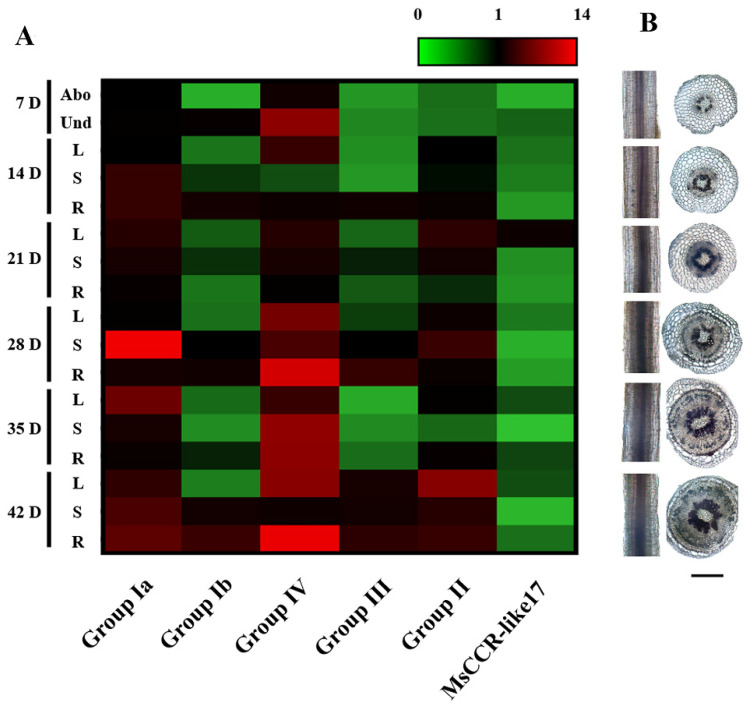
The expression profiles of *MsCCR*/*MsCCR-like* genes in different tissues at 1–6 weeks of different growth period. Gene expression (**A**) and phloroglucinol histochemical staining (**B**) were analyzed at different parts after 7, 14, 21, 28, 35, and 42 days (D) of growth. Data were from three independent experiments with at least three replicates for each. *Actin2* was used as internal reference, and the relative expression of each gene was further compared to the sample of the above ground part after 7 days of growth. Abo: above ground; Und: underground; L: leaf; S: stem; R: root. Bar = 1 mm.

RT-qPCR was then performed to examine the role of *MsCCR/MsCCR-like* genes in alfalfa during different developmental stages. According to the specificity of CDS sequences, 6 pairs of specific primers were obtained, for example, Group Ia (*MsCCR1–4*), Group Ib (*MsCCR5–7*), Group IV (*MsCCR-like8–11, 23*), Group III (*MsCCR-like5–7*), Group II (*MsCCR-like1–4*), and *MsCCR-like17*. As shown in Figure 5A, the transcripts of *MsCCR1–4* (Group Ia) were upregulated, especially in the shoot after 28 days of growth. Meanwhile, the transcripts of *MsCCR5–7* (Group Ib) did not change considerably, with higher levels underground than aboveground. Unexpectedly, transcripts of genes in Group IV showed more abundance than Group Ia, and increased expression with the development process was also shown. In addition, the expression pattern of genes in Group III was similar to Group Ib, and Group II was similar to Group Ia. The transcript of *MsCCR-like17* exhibited slight induction, especially in leaves. Meanwhile, histochemical staining was employed to explore the lignin levels in the alfalfa plant stem and root. The coloration gradually deepened with the development process, especially after 28 days of growth.

### 2.5. The Expression Patterns of MsCCRs/MsCCR-likes under Abiotic Stresses and Hormone Treatment

**Figure 6:**
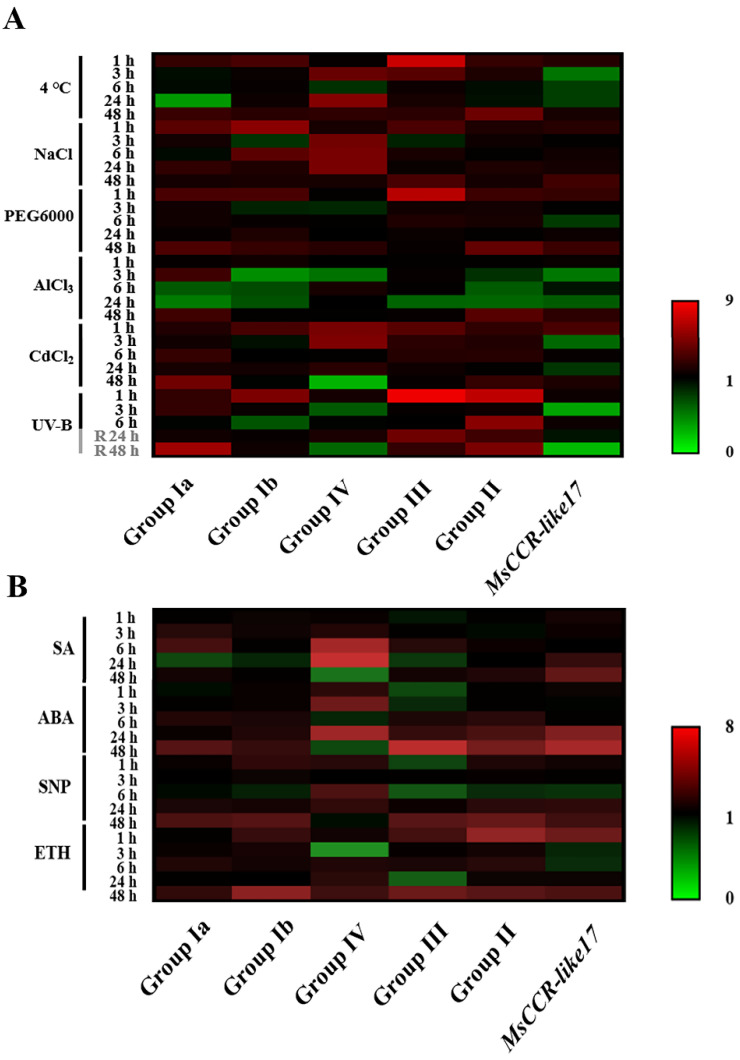
The expression profiles of *MsCCR*/*MsCCR-like* genes under different treatments and different times in *M. sativa*. Seven-day-old alfalfa seedlings were used for abiotic stresses (**A**) and phytohormone (**B**) treatment. After 1, 3, 6, 24, and 48 h of 4 °C, NaCl (100 mM), PEG6000 (15%), AlCl_3_ (150 μM), CdCl_2_ (100 μM), SA (50 mg/L), ABA (50 mg/L), SNP (80 μM), and ETH (500 μM) treatment, the transcripts were analyzed by RT-qPCR. UV-B (1 J·m^−2^·h^−1^) was treated for 6 h, followed by 12 h of recovery (in grey). Data were from three independent experiments with at least three replicates for each. *Actin2* was used as internal reference, and the relative expression of each gene was further compared to control samples at the same time point under each treatment.

Further experiments were performed to detect the response of *MsCCR/MsCCR-like* genes under various stresses and hormone treatments. As shown in Figure 6A, with cold (4 °C), NaCl, PEG6000, AlCl_3_, and CdCl_2_ treatment, the transcript levels of genes in Group Ia and Group II were slightly increased at early (1 h) and late stage (48 h), but downregulated (especially under Al stress) at 6 and 24 h of treatment. Meanwhile, the transcripts of genes in Group Ib and Group III showed a similar trend but it was more obvious under 4 °C and NaCl treatments. The genes in Group IV were mainly induced at 3–24 h with 4 °C and NaCl treatments, and there was strong induction at 1–3 h and reduction at 48 h. The transcript of *MsCCR-like17* showed decreased expression after 3–24 h of 4 °C, NaCl, PEG6000, AlCl_3_, and CdCl_2_ treatments. After 1 h of ultraviolet-B (UV-B) treatment and subsequent recovery period, genes in Group Ia/Ib/II/III were upregulated, while *MsCCR-like17* and genes in Group IV showed a downregulated trend during stress treatment and recovery periods.

After exogenous salicylic acid (SA) treatment, the transcripts of genes in Group Ia/Ib/III were slightly upregulated at 6 h but decreased after 24 h of treatment (Figure 6B). The abscisic acid (ABA), nitric oxide donor sodium nitroprusside (SNP), and ethephon (ETH) treatments showed similar trends with the upregulated expression of genes in Group Ia/Ib/II at the late stage (48 h) of treatment. The genes in Group III showed a downregulated trend before 24 h of ABA, SNP, and ETH treatment but were upregulated after 48 h of treatments. In addition, the transcripts in Group IV were increased during the first 24 h but decreased after 48 h of SA, ABA, and SNP treatments, and they showed an obvious decrease at the 3 h point of the ETH treatment. While the expression of *MsCCR-like17* was enhanced at the late stage (24–48 h) of treatment.

### 2.6. Cloning, Heterologous Expression, and Enzymatic Assay of MsCCR1 and MsCCR-like1 Gene

To illustrate the biochemical functions of MsCCRs, we cloned *MsCCR1* (in Group I) and *MsCCR-like1* (in Group II) gene. Heterologous expression of the His-tagged MsCCR1 and MsCCR-like1 was successfully obtained from *E. coli* by 0.2 mM isopropyl-*β*-_D_-thiogalactopyranoside (IPTG) induction at 16 °C. Recombinant MsCCRs were purified with Ni^2+^ affinity chromatography, followed by molecular masses analysis through SDS-PAGE. The molecular masses of the MsCCR1 exhibited about 37.21 kDa, and MsCCR-like1 was about 33.61 kDa, which is consistent with their theoretical molecular mass (Figure S2).

Furthermore, the kinetic parameters of recombinant MsCCR1 and MsCCR-like1 to different substrates, including feruloyl-, *p*-coumaroyl-, caffeoyl-, and sinapoyl-CoAs, were investigated, respectively. As shown in Table 1, MsCCR1 catalyzed feruloyl-CoA, *p*-coumaroyl-CoA, and sinapoyl-CoA, but it had the lowest Michaelis constant value (*K_m_*) for sinapoyl-CoA. In addition, the catalytic efficiency (*K_cat_*/*K_m_*) of MsCCR1 for sinapoyl-CoA was more than twice that for feruloyl-CoA and *p*-coumaroyl-CoA. Correspondingly, MsCCR-like1 catalyzed *p*-coumaroyl-CoA and caffeoyl-CoA, with similar *K_m_* and *K_cat_*/*K_m_*. The different characters in catalyzing suggest their diverse functions in lignin synthesis.

### 2.7. Three-Dimensional Structure Analysis of CCRs in M. sativa and Other Plants

**Figure 7:**
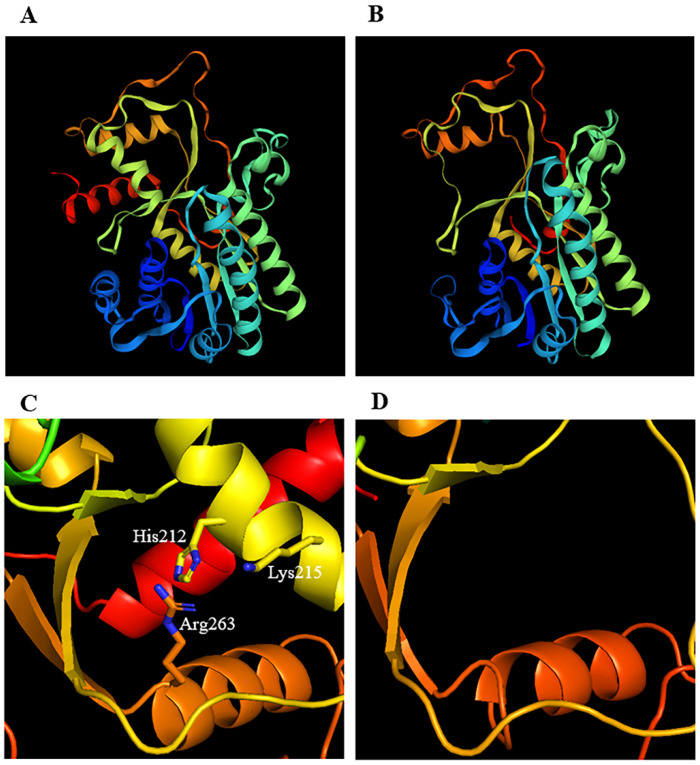
The predicted three-dimensional structure of MsCCR1 (**A**) and MsCCR-like1 (**B**), and the detailed structure in the C-terminal end of MsCCR1 (**C**) and MsCCR-like1 (**D**). The spirals, arrows, and lines indicate the α-helices, β-sheets, and turns and coils, respectively. The sticks indicate catalytic triads H212(X)2K215R263 with corresponding.

The three-dimensional structure models of MsCCR1 and MsCCR-like1 proteins in the bona fide clade were constructed (Figure 7A,B). It can be seen that a part of the MsCCR-like1 in the C terminal is missing, which including the substrate-binding motifs H212(X)2K215R263 (Figure 7C,D). Furthermore, three-dimensional structure models of some representative members MsCCR5, MsCCR-like7/11/17/21, and typical CCR protein in other species, including MtCCR, AtCCR1/2, TaCCR2, EgCCR, PtCCR, LlCCR1, ZmCCR2, and PaCCR2, were also constructed (Figure S3). The structures of MsCCR1, MsCCR5, and the identified CCRs in other plants share high similarities, but MsCCR-likes exhibited structural changes within them in some parts of the protein (Figure S4). In addition, the alignment of the above CCR/CCR-like proteins showed that some key amino acid residues are missing in MsCCR-likes compared to CCRs. These results suggest that the catalytic function of the MsCCR-likes might be different from that of MsCCRs.

## 3. Discussion

CCR plays a central role in lignin biosynthesis, and it is considered to be a biotechnological target in lignocellulosic biomass engineering [40]. Meanwhile, it is also the target to decrease lignin content in alfalfa because of its enormous influence on forage digestibility [41]. However, little is known about the CCR gene family in the widely cultivated forage *M. sativa*. In this study, we identified 7 *MsCCR* genes with the location at chromosomes 2.1–2.4 and 4.1–4.3 in *M. sativa* (Figure 1 and Figure S1; Table S1). The proteins encoded by *MsCCR1*–*4* (with the same protein length and pI) and *MsCCR5*–*7* (with the same protein length and pI) are homologous to functionally characterized MtCCR1 and MtCCR1-2, respectively (Table S1; [34,42]). The deficiency of the CCR gene in chromosome 4.4 suggested chromosomal fragment loss events during evolution. In addition, we also identified 23 *MsCCR-likes* with partially altered motif KNWYCYGK. The phylogenetic trees showed they are clustered together with a closed relationship to *AtCCR-like* and *MtCCR-likes* (Figure 2).

Diverse CCR gene families have been reported in some plant species. In *Arabidopsis*, 11 putative CCR genes have been identified [43]. In addition, there are 11 CCRs in *Populus tomentosa*, 33 in *Oryza sativa*, 10 in *Eucalyptus grandis*, 31 in pear (*Pyrus bretschneideri Rehd*.), and 9 in *Poplus trichocarpa* [7,24,33,44,45]. The CCR member in most diploid plants is almost half in the tetraploid plant such as alfalfa and some diploid plants with whole-genome duplication events such as Chinese white pear. In addition, this is also the reason that there have been many tandem duplication events during alfalfa evolution, which can be concluded from the synteny analysis of *CCR/CCR-like* genes in *M. sativa* and *M. truncatula* (Figure S1).

The *cis*-element predicted in the promoter of *MsCCR/MsCCR-like* genes suggested the involvement of *MsCCR/MsCCR-like* in plant development (MYB/MYC), phytohormone response (ABRE), and stresses response, such as light (GT1-motif), anaerobic induction (ARE), and MYB-involved abiotic stress responses (Figure 4; [46,47]). Further expression profiling showed that *MsCCR1–4* (Group Ia) and *MsCCR-like1–4, 8–11, 23* (Groups II and IV) are mainly involved in lignin synthesis during the development process (Figure 5). The lignin content and CCR gene expression were gradually increased in tobacco during maturation [48]. Similarly, the transcripts of *CsCCR* were raised during the stem development of the tea plant, and they showed higher levels in “Fudingdabai” compared to “Suchazao”, with the results of higher lignin accumulation in “Fudingdabai” compared to “Suchazao” [49]. The transcription of lignin-synthesis-related genes can be regulated by transcript factors such as MYBs [50,51]. However, in the expression of *MsCCR5–7* (Group Ib) and some *MsCCR-likes* (Group III and *MsCCR-like17*), a few changes even decreased during the 28 days of plant development (Figure 5). This may be because of the different *cis*-element in the promoter of these genes compared to that in Group Ia, II, and IV (Figure 4). Interestingly, some researchers argue that not all members of the CCR families are involved in lignin synthesis after a long period of evolution [52].

Meanwhile, the expression of *MsCCR5–7* (Group Ib), *MsCCR-like5–7* (Group III), and *MsCCR-like8–11, 23* (Group IV) was upregulated with varying degrees after chilling, salinity, drought, heavy metals, and UV-B stresses (Figure 6). Several reports have shown that CCR participated in plants coping with environmental stresses. The early response genes to salt stress in the root of melon seedlings include *CCR* and transcript factor *MYB* [53]. *CCR11* was upregulated under salt and water-deficient conditions in *Populus trichocarpa* [54]. In addition, the expression patterns of *MsCCR1–4* and *MsCCR5–7* were different during development and stress response (Figure 5 and Figure 6). This is consistent with a further report that *CCR1* is involved in lignification during the development process, while *CCR2* is involved in stress responses in *Arabidopsis* [28]. In addition, the loss function of *MtCCR1* showed deficiency in plant growth, but the mutation of *MtCCR2* does not exhibit inhibited plant growth [34,42]. The function of *MsCCR5–7* (homologous to *MtCCR2* in [34]) and most *MsCCR-likes* might be plant defense against biotic and abiotic stresses by regulating lignin biosynthesis [55,56]. These diverse function and expression profiles of CCRs might be related to their duplication during evolution [57].

The expression of *MsCCRs* and *MsCCR-likes* (in particular) exhibited different induced levels under phytohormone SA, ABA, SNP, and ETH treatments (Figure 6). These findings indicated that CCR participated in phytohormone-mediated development, biotic stress, and abiotic stress responses. For example, the ethylene response factor AP2/ERF Ii049 mediated methyl jasmonate, SA, and ABA response by regulating CCR-associated lignin synthesis [58]. In addition, the expression levels of CCRs after ABA treatment are in accordance with the results of the ABRE element in the promoter of CCRs in alfalfa (Figure 4 and Figure 6). The underlying pathway might include some transcript factors such as MYBs and NACs [23,56,59]. Furthermore, it was found that ETH-treated strawberry showed higher hemicelluloses, cellulose, and neutral sugars than the control [60]. Al-induced pectin and hemicellulose accumulation can be alleviated by SNP treatment in rice root cells [61]. Although the effects of lignin content on stress resistance and feed quality are contradictory, these results suggested the possible role of other composites in the cell walls to solve this contradiction, in other words, with reduced lignin and enhanced resistance.

To further illustrate the functions of the MsCCRs and MsCCR-likes, we cloned and expressed recombinant MsCCR1 and MsCCR-like1, followed by the biochemical analyses (Table 1; Figure S2). The MsCCR1 showed catalytic ability with the substrates feruloyl-CoA, *p*-coumaroyl-CoA, and sinapoyl-CoA, which is similar to the findings in EgCCR, AtCCR1, and PhCCR1 [62,63,64]. Meanwhile, the substrates *p*-coumaroyl-CoA and caffeoyl-CoA can be catalyzed by MsCCR-like1, suggesting that MsCCR-likes also play a role in lignin synthesis. Combined with the promoter analysis and expression profile (Figure 4 and Figure 6), the functions of CCR-like might be linked to plant defense response [65]. Chao et al. [38] point out that H2O2(X)2K205 is a crucial motif that can be used to distinguish between CCRs and CCR-like proteins. In this text, the results from three-dimensional structure models showed the primary difference is the missing of the H212(X)2K215R263 motif and the C-terminal end in MsCCR-like1 protein (Figure 7), which also can be found in the results of protein alignment (Figure 3). Prasad et al. [1] concluded that the ARG51, ASN52, ASP54, and ASN58 in CCRs are critical residues for feruloyl binding. In this study, the motif RNXD(X)3N is found in MsCCR1 (NADP specificity box in Figure 3) but not in MsCCR-like1 (with QQYGEXXX instead). Pan et al. [64] found that residues Ile124, Gly125, Val185, Leu186, Ala220, and Tyr284 formed a Ph-CCR1 binding pocket for the phenolic ring. The MsCCR1 is in possession of I134, G135, V195, L196, A206, and Y275 residues, which consist of the reported CCR proteins, but not in MsCCR-like1 and other MsCCR-likes (Figures S3 and S4). These results highlight the potential use of the CCRs in alfalfa genetic engineering, as well as pharmacologic decreased CCR gene expression by specific chemicals. On the contrary, environmental-stresses-induced CCR gene expression might be helpful for plant defense but might have a negative effect on forage quality. Therefore, the overall quality should also be considered when the genetic approach was implemented targeting lignin-reduced plants. Furthermore, it will be very interesting to study the function of CCR-likes and to compare the structural and functional differences between CCR and CCR-like in future work.

## 4. Materials and Methods

### 4.1. Plant Growth and Treatments

Sterilized alfalfa seeds (*M. sativa* L. Victoria, Clover Seed & Turf Co., Beijing, China) were germinated at 25 °C for 1 d, and then uniform seeds were selected and transferred to the illuminating incubator in quarter-strength Hoagland’s solution [66]. Five-day-old seedlings were used for common environmental stresses and plant hormone treatments. NaCl was used for salinity stress at 100 mM [67]; PEG6000 was used for mimicking drought stress at 15% (*w*/*v*) [68]; AlCl_3_ was used at 150 μM [69]; and CdCl_2_ was used at 100 μM [66]. UV-B treatment was performed using the methods described by Xie et al. [70], 1 J·m^−2^·h^−1^ treated for 6 h and recovered for another 42 h. Salicylic acid (SA) was used at 50 mg/L [71]; abscisic acid (ABA) was used at 50 mg/L [72]; nitric oxide donor sodium nitroprusside (SNP) was used at 80 μM [61]; and ethephon (ETH) was used at 500 μM immediately after preparation [60]. All the reagents were purchased from Sigma-Aldrich (St. Louis, MO, USA).

### 4.2. MsCCR Identification and Classification

For *MsCCRs* gene determination, a local protein database was established by using DNATOOLS software with alfalfa (*M. sativa* L.) genomic data [36,73]. To identify candidates of the *CCR* family, sequences of 2 CCRs from *Arabidopsis thaliana* (At1G15950 and At1G80820) and 6 CCRs from *Medicago truncatula* (MTR_2g104960, MTR_4g006940, MTR_5g029990, MTR_6g406250, MTR_2g028620, and MTR_4g009040) were used for BLASTp and BLASTn (E-value ≤ 0.001; Identities ≥ 80%; obtained from NCBI: www.ncbi.nlm.nih.gov, accessed on 30 April 2021) analysis. When comparing, the single exon similarity of each candidate must be above 85%, and the exon deletion number must be less than or equal to 2. After that, all the candidates were blasted against CDS/mRNA database, and a score greater than 80 was determined as a member of *MsCCR* gene family. Finally, they were confirmed by sequence alignment against the protein database. Regions of resulting signature motifs were identified by a MEGA-X ClustalW sequence alignment, cutoff rate = 30%. If a candidate contains the CCR signature motif “(KNW)YCYGK”, it will be named *MsCCR*, or it will be named *MsCCR-like*. The pI and MW of amino acids were calculated using ExPASy Compute pI/MW [74].

### 4.3. Motif Analysis and Gene Structure Visualization

Conversed motifs were found with the widely used online tool Multiple Em for Motif Elicitation (MEME; [75]) [76,77]. Detailed settings are below: motif width is between 6 aa and 50 aa; the maximum motif numbers are set to 20; running under classic model. GSDS online tool [78] was used to visualize exon-intron structure.

### 4.4. Multiple Sequence Alignment and Phylogenic Analysis Evolutionary Analysis

The phylogenetic tree was constructed with the neighbor-joining model, JTT method (bootstrap = 400) by MEGA-X [79], and iTOL [80] for decoration. Similarity analysis was conducted through BioEdit [81]. All the sequences used for the phylogenetic tree and alignment are listed in Table S3.

### 4.5. Cis-regulatory Element Analysis and 3D Structure Prediction

First, 2000 bp upstream was extracted from the initiation codon of each gene to ensure that they contained the promotor region. These sequences were used for cis-regulatory element analysis using PlantCARE [82]. Prediction of the 3D structure was conducted by SWISS-MODEL [83] for modeling (GMQE ≥ 0.8) and PyMOL [84] for the exhibition of detailed structure.

### 4.6. Collinearity Analysis and Chromosome Localization

Collinearity analysis was conducted by TBtools [85] One-step MCScanX, and visualized by TBtools Dual Systeny Plot. Chromosome localization was completed by TBtools Gene Location Visualize from GTF/GFF.

### 4.7. RNA Isolation and RT-qPCR Analysis

For the total RNA isolation, 500 mg of alfalfa tissues (leaf, stem, and root) under different stages or after different treatments was homogenized and further extracted by using the RNAiso Plus kit (Takara, Japan, Dalian). Five micrograms of total RNA was used for reverse transcription with the HiScript III 1st Strand cDNA Synthesis Kit (Vazyme, Nanjing, China). qPCR was carried out on an Applied Biosystems™ QuantStudio™ 5 (ABI, Los Angeles, CA, USA). The reaction contains 2× ChamQ Universal SYBR qPCR Master Mix (Vazyme, China, Nanjing) 10 μL, primer F/R (10 μM) 0.4 μL, template 200 ng, and ddH_2_O makes up to 20 μL. Relative expression levels were calculated by using the 2^−ΔΔCT^ method with *Actin2* used as the internal reference. The primer sequences are listed in Table S4.

### 4.8. Lignin Content and Staining Analysis

The main stem was sliced by hand and soaked in 2% phloroglucin (in 95% ethanol) for 5 to 10 s, then treated with 12% muriatic acid for 5 to 10 s until lignin turns red. The time was extended to 20 to 30 s when treating the whole root.

### 4.9. Cloning, Expression, and Purification of Recombinant MsCCR

The target sequences were cloned from the cDNA template. Plasmid *pET-28α* was used to construct the expression vector. Constructed vectors were stored in *Escherichia coli* DH5α. The vector was transferred into *E. coli* BL21 and cultured in Luria–Bertani (LB) medium 37 °C 210 rpm. The recombinant proteins were induced at 16 °C 150 rpm with 200 μM isopropy-β-_D_-thiogalactoside (IPTG) in LB medium for at least 5 h or overnight when OD_600_ = 0.6–0.8. Induced BL21 was centrifuged and resuspended in 1× PBS. The cells were disrupted on ice by a sonic dismembrator with 100 μM phenylmethylsulfonyl fluoride (PMSF). After centrifuging at 8000 rpm 4 °C for 20 min, the liquid was filtered (0.45 μm filter) in BBI™ Ni-NTA 1 mL (Prepacked Gravity Column), then eluted with imidazole of different concentrations (20 mM, 50 mM, 100 mM, 250 mM, or 500 mM) in LE buffer. The His-tagged MsCCR proteins were eluted with 100 mM imidazole. The His-tagged MsCCR proteins were eluted with 100 mM imidazole. The purification of obtained proteins was valued by Western blot using an anti-His tag antibody.

### 4.10. CCR Activity and Enzyme Kinetic Parameter Analysis

The protein eluted by 100 mM imidazole was dialyzed in 1 × PBS with 1 mM dithiothreitol (DTT), magnetic stirring on ice for 3 h. BCA Protein Assay Kit (Takara, China, Dalian) was used to detect the protein content of dialyzed enzymes. For enzymatic analysis, a 500 μL reaction was used, containing the following content: enzyme 20 μg, NADPH 160 μM, substrate (feruloyl-CoA, *p*-coumaroyl-CoA, caffeoyl-CoA, and sinapoyl-CoA) (ZZBIO CO., LTD, China, Shanghai) 30–150 μM, 1 × PBS to 500 μL. The decrease of A_366_ was recorded, and changed the concentration of different substrates. The parameters *K*_m_, *V*_max_, and *K*_cat_ will be calculated by *V*_0_/[S] − *V*_0_ plots.

## 5. Conclusions

The present study identified 30 CCRs in *M. sativa* genome, and further analyzed the phylogenetics and evolution of CCR family proteins. Seven *MsCCR* genes were located on seven chromosomes in alfalfa, which are homologous to two CCR genes in *M. truncatula*. Meanwhile, 23 *MsCCR-like* genes were categorized into four subfamilies. The expression patterns in alfalfa seedlings at different growth stages suggested that *MsCCR* genes play an important role in cell wall lignification. In addition, both *MsCCR* and *MsCCR-like* genes responded to environmental stresses and hormone treatment, which is consistent with the *cis*-elements in their promoters. Furthermore, the biochemical and three-dimensional structure model analysis of recombinant MsCCR1 and MsCCR-like1 suggested the importance of some motifs for catalytic activities under the specific substrates.

## Figures and Tables

**Table 1:** Kinetic analysis of MsCCR1 and MsCCR-like1.

Symbols	Feruloyl-CoA		*p*-Coumaroyl-CoA		Caffeoyl-CoA		Sinapoyl-CoA	
	*K*_m_	*K*_cat_/*K*_m_	*K*_m_	*K*_cat_/*K*_m_	*K*_m_	*K*_cat_/*K*_m_	*K*_m_	*K*_cat_/*K*_m_
MsCCR1	45.20 ± 11.42 ab	2.09 ± 0.18 B	24.22 ± 4.51 bc	2.58 ± 0.11 B	ND	ND	10.57 ± 1.25 c	5.72 ± 0.36 A
MsCCR-like1	ND	ND	44.14 ± 7.12 ab	1.09 ± 0.19 C	48.16 ± 1.75 a	1.09 ± 0.11 C	ND	ND

*K*_m_: Michaelis constant (μM); *K*_cat_/*K*_m_: catalytic constant/Michaelis constant (μM min^−1^); “ND”: not detectable. Data are mean ± SE of three independent experiments. Different letters indicate significant differences at *p* < 0.05 according to Duncan’s multiple range test.

## Data Availability

All data are contained within the article and supplementary files.

## Supplementary Materials

The following supporting information can be downloaded at: https://www.mdpi.com/article/10.3390/ijms23147762/s1.
