# Supplementary material for: Characterization, Expression Profiling, and Biochemical Analyses of the Cinnamoyl-CoA Reductase Gene Family for Lignin Synthesis in Alfalfa Plants
Source: Int J Mol Sci. 2022 Jul 14;23(14):7762. doi: 10.3390/ijms23147762 (PMC9316543; doi:10.3390/ijms23147762)
Supplement: Supplementary file 1 [file ijms-23-07762-s001.zip › ijms-1805880-supplementary.pdf]

Figure S1

A

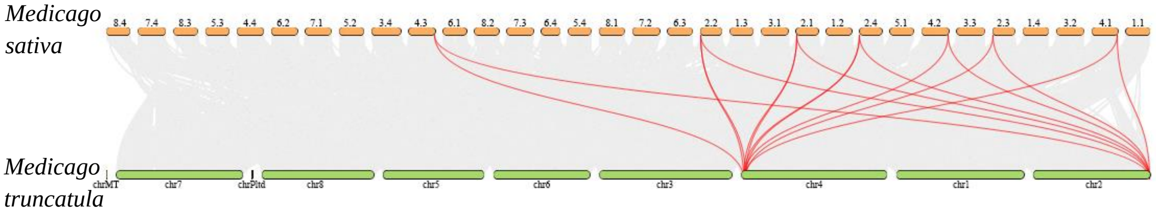

B

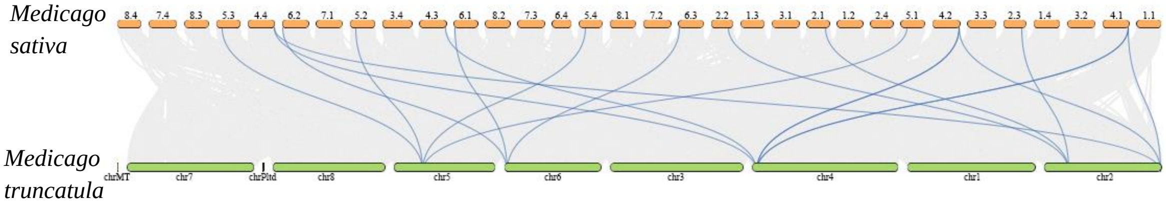

Figure S1. The chromosome location and synteny analysis of *CCR*/*CCR-like* genes in *M. sativa* and *M. truncatula*. Gray lines in the background indicate the collinear blocks within *M. sativa* and *M. truncatula*. The red (**A**, *MsCCRs*) or blue (**B**, *MsCCR-likes*) curves highlight the syntenic *CCR*/*CCR-like* gene pairs.

Figure S2

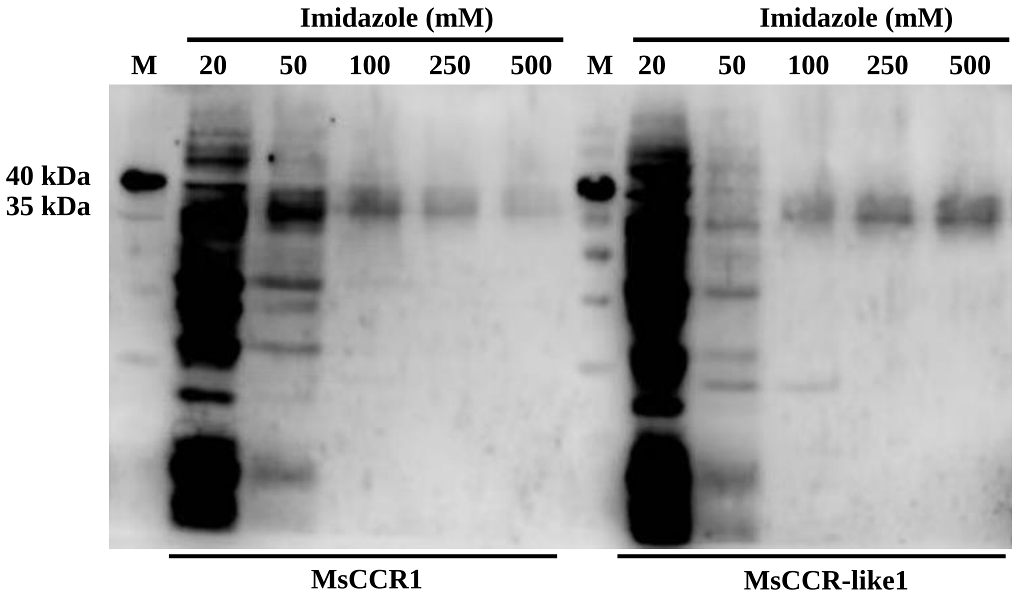

Figure S2. Western blot profile of recombinant MsCCR1 and MsCCR-like1. M: protein marker.

Figure S3

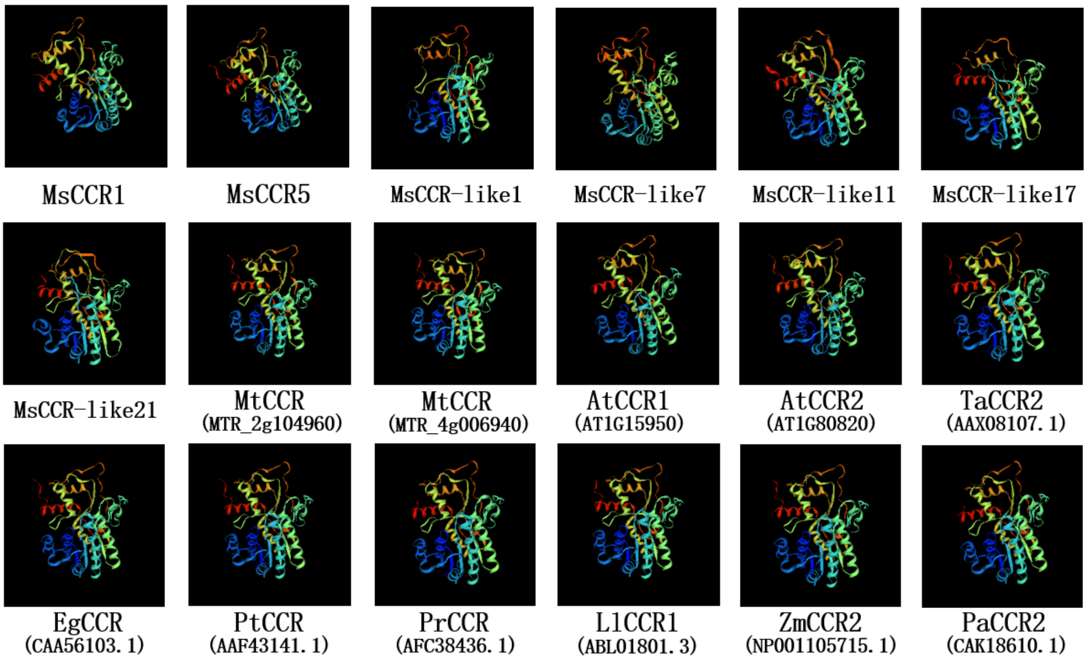

Figure S3. Predicted three-dimensional structures of MsCCR/MsCCR-likes and other bona fide CCRs.

Figure S4

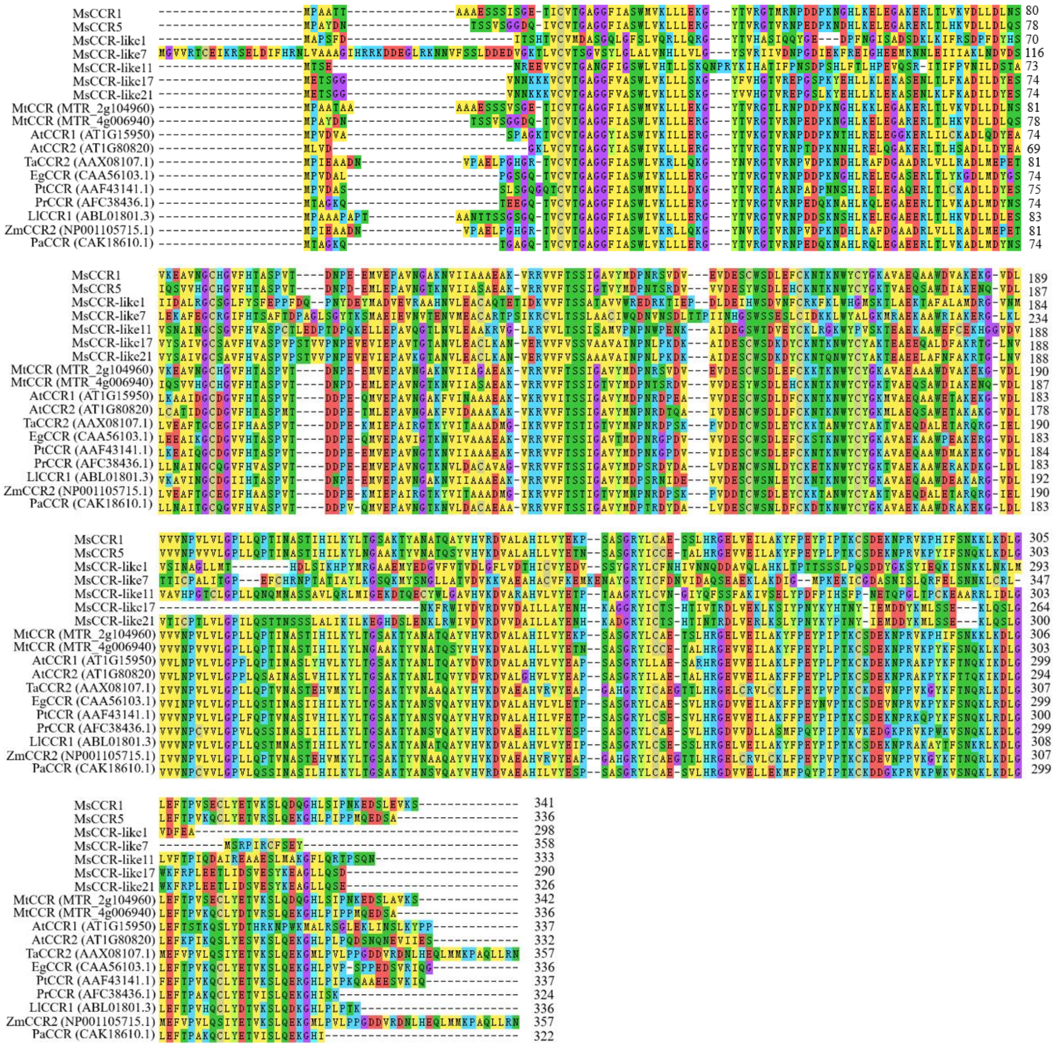

Figure S4. Alignments of the MsCCR/MsCCR-like proteins and other bona fide CCRs.

Table S1. Properties of *CCR* genes and the predicted CCR proteins in *M. sativa*

Gene Name	Chromosome Location	Homologous Gene in *M. truncatula*	Locus Tag of Homologous Gene	pI	Theoretical MW (Da)	Protein Length
MsCCR1	chr2.1: 1039704 - 1044022	*MtCCR1*	MTR_2g104960	5.90	37205.54	341
MsCCR2	chr2.2: 1307658 - 1311376	*MtCCR1*	MTR_2g104960	5.90	37187.51	341
MsCCR3	chr2.3: 140748 - 144403	*MtCCR1*	MTR_2g104960	5.90	37187.51	341
MsCCR4	chr2.4: 712408 - 716186	*MtCCR1*	MTR_2g104960	5.90	37187.51	341
MsCCR5	chr4.1: 88441560 - 88444191	*MtCCR1-2*	MTR_4g006940	5.89	37271.43	336
MsCCR6	chr4.2: 92674173 - 92676815	*MtCCR1-2*	MTR_4g006940	5.89	37271.43	336
MsCCR7	chr4.3: 88471169 - 88473810	*MtCCR1-2*	MTR_4g006940	5.89	37271.43	336
MsCCR-like1	chr5.4: 20239570 - 20242878	*MtCCR-like SNL6*	MTR_5g029990	5.00	33606.88	298
MsCCR-like2	chr5.3: 18940776 - 18944245	*MtCCR-like SNL6*	MTR_5g029990	5.00	33609.87	298
MsCCR-like3	chr5.2: 18815018 - 18818013	*MtCCR-like SNL6*	MTR_5g029990	5.00	33575.85	298
MsCCR-like4	chr5.1: 20561135 - 20564090	*MtCCR-like SNL6*	MTR_5g029990	5.00	33589.88	298
MsCCR-like5	chr6.1: 1696762 - 1702383	*MtCCR-like SNL6-2*	MTR_6g406250	7.48	39783.82	358
MsCCR-like6	chr6.2: 1718684 - 1724261	*MtCCR-like SNL6-2*	MTR_6g406250	7.48	39849.89	358
MsCCR-like7	chr6.3: 1881162 - 1886448	*MtCCR-like SNL6-2*	MTR_6g406250	7.48	39852.90	358
MsCCR-like8	15006: 7272 - 10850	*MtCCR1-3*	MTR_2g028620	5.89	36599.74	333
MsCCR-like9	chr2.3: 60709715 - 60713293	*MtCCR1-3*	MTR_2g028620	5.89	36599.74	333
MsCCR-like10	chr2.1: 62319777 - 62323333	*MtCCR1-3*	MTR_2g028620	5.89	36623.79	333
MsCCR-like11	chr2.2: 59055829 - 59059406	*MtCCR1-3*	MTR_2g028620	5.94	36642.83	333
MsCCR-like12	chr4.4: 89890905 - 89894155	*MtCCR1-4*	MTR_4g009040	5.54	35926.18	325
MsCCR-like13	chr4.3: 86683346- -86686357	*MtCCR1-4*	MTR_4g009040	5.41	36184.27	326
MsCCR-like14	chr4.2: 91110498 - 91113703	*MtCCR1-4*	MTR_4g009040	5.43	36043.25	326
MsCCR-like15	chr8.3: 39385671 - 39389109	*MtCCR1-4*	MTR_4g009040	5.78	36175.44	325
MsCCR-like16	chr8.2: 38259260 - 38262698	*MtCCR1-4*	MTR_4g009040	5.78	36175.44	325
MsCCR-like17	chr4.2: 90649870 - 90653251	*MtCCR1-4*	MTR_4g009040	5.93	32349.96	290
MsCCR-like18	chr4.1: 85995762 - 85999167	*MtCCR1-4*	MTR_4g009040	6.26	36075.51	325
MsCCR-like19	chr2.4: 48916854 - 48920360	*MtCCR1-4*	MTR_4g009040	5.52	35988.13	326
MsCCR-like20	chr2.2: 46570633 - 46574128	*MtCCR1-4*	MTR_4g009040	5.94	35874.04	325
MsCCR-like21	chr4.1: 86755522 - 86759200	*MtCCR1-4*	MTR_4g009040	5.95	36177.50	326
MsCCR-like22	chr2.3: 48445104 - 48452492	*MtCCR1-4*	MTR_4g009040	5.94	35861.04	325
MsCCR-like23	15007: 40384 - 43962	*MtCCR1-3*	MTR_2g028620	5.89	36599.74	333

Table S2. Motif compositions of CCR proteins from *M. sativa*.

Motif No.	Width	Sequences
Motif 1	41	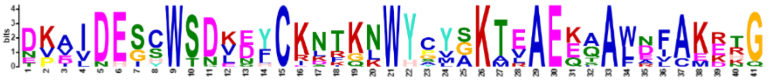
Motif 2	42	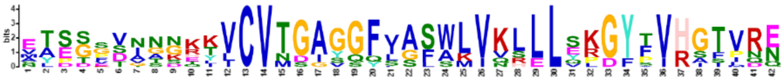
Motif 3	41	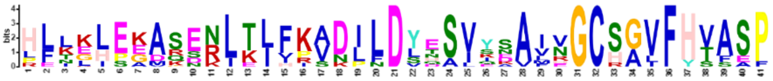
Motif 4	21	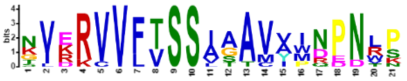
Motif 5	21	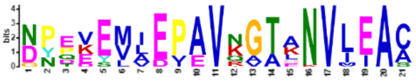
Motif 6	21	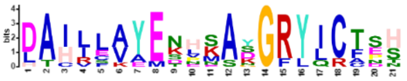
Motif 7	25	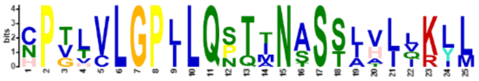
Motif 8	35	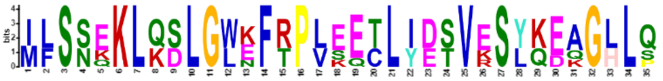
Motif 9	29	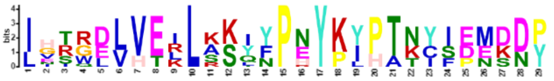
Motif 10	15	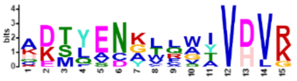
Motif 11	21	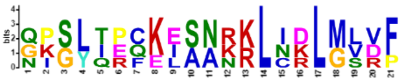
Motif 12	29	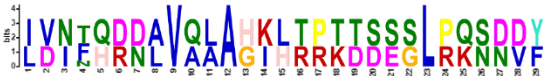
Motif 13	15	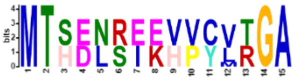
Motif 14	6	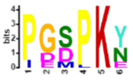
Motif 15	29	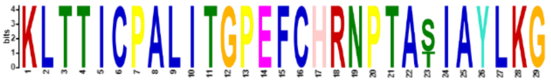
Motif 16	6	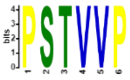
Motif 17	6	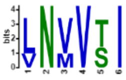
Motif18	8	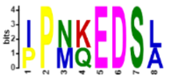
Motif 19	11	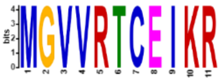
Motif 20	8	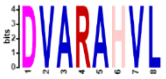

Table S3. Sequences of the CCR proteins in important bona fide CCRs.

Protein names	IDs	Sequences
AtCCR1	At1G15950	mpvdvaspagktvcvtgaggyiaswivkillergytvkgtvrnpddpknthlrgleggkerlilckadlqdyealkaai<br>dgcdgvfhtaspvtddpeqmvepavngakfvinaaaeakvkrvvitssigavymdpnrdpeavvdescwsdldfc<br>kntknwycygkmvaeqaawetakekgvdlvvlnpvlvlgpplqptinaslyhvlkyltgsaktyanltqayvdvrdv<br>alahvlvyeapsasgryllaesarhrgevveilaklfpeyplptkckdeknprakpykftnqkikdlgleftstkqslydta<br>sqesvengikigs
AtCCR2	At1G80820	mlvdgklvcvtgaggyiaswivklllergytvrgtvrnptdpknnhlrelqgakerltlhsadlldyealcatidgcdgvf<br>htaspmtddpetmlepavngakfvidaaakakvkrvvftssigavymnpnrdtqaivdencwsdldfckntknwyc<br>ygkmlaeqsawetakakgvdlvvlnpvlvlgpplqsainaslvhilkyltgsaktyanltqvyvdvrdvalghvlvyea<br>psasgryilaetalhrgevveilakffpeyplptkcsdeknprakpykfttqkikdlglefkpikqslyesvkslqekghlp<br>lpqdsnqneviies
PtCCR	AAF43141.1	mpvdasslsgqgqtcvtgaggfiaswmvklldkgytvrgtarnpadpnnshlrelegaqerltlckadlldyeslkeai<br>qgcdgvfhtaspvtddpeemvepavngtknviiaaaeakvrrvvftssigavymdpnkgpdvvidescwsdlefck<br>ntknwycygkavaeqaawdmakekgvdlvvvnpvlvlgplfqptvnasivhilkyltgsaktyansvqayvhvrdv<br>alahilvfetpsasgrylcsesvlhrgevveilakffpeypiptkcsdeknprkqpykfsnqklrdlgfeftpvkqclyetv<br>kslqerghlpipkqaaeesvkiq
PtrCCR2	ACC63879.1	mpvdasslsgqgqticvtgaggfiaswmvklldkgytvrgtarnpadpknshlrglegaeerltlckadlldyeslkeai<br>qgcdgvfhtaspvtddpeemvepavngtknviiaaaeakvrrvvftssigavymdpnkgpdvvidescwsdlefck<br>ntknwycygkavaeqaawdmakekgvdlvvvnpvlvlgpllqptvnasithilkyltgsaktyansvqayvhvrdv<br>alahilvfetpsasgrylcsesvlhrgevveilakffpeypiptkcsdeknprkqpykfsnqklrdlgfeftpvkqclyetv<br>kslqekghlpipkqaaeeslkiq
EgCCR	CAA56103.1	mpvdalpgsgqtvcvtgaggfiaswivklllergytvrgtvrnpddpknghlrelegaserltlykgdlmdygsleeaik<br>gcdgvvhtaspvtddpeqmvepavigtknvivaaaeakvrrvvftssigavtmdpnrgpdvvvdescwsdlefckst<br>knwycygkavaekaawpeakergvdlvvinpvlvlgpllqstinasiihilkyltgsaktyansvqayvhvkdvalah<br>vlvletpsasgrylcaesvlhrgdvveilakffpeynvptkcsdevnprvkpykfsnqklrdlgleftpvkqclyetvksl<br>qekghlpvpsppedsvriqg
PhCCR1	AHX56186.1	mrsvsgqvvcvtgaggfiaswlvkillekgytvrgtvrnpddpknghlrelegakerltlckadlldyqslreaingcdg<br>vfhtaspvtddpeqmvepavigtknvinaaaeanvrrvvftssigavymdpnrdpetvvdetcwsdpdfckntknw<br>ycygkmvaeqaaweeakekgvdlvvinpvlvqgpllqttvnasvlhilkyltgsaktyansvqayvdvkdvalahill<br>yetpeasgrylcaesvlhrgdvveilskffpeypiptkcsdvtkprvkpykfsnqklkdlgleftpvkqclyetvkslqek<br>ghlpiptqkdepiiriqp
LlCCR1	ABL01801.3	mpaaapaptaanttssgsgqtvcvtgaggfiaswivklllergytvrgtvrnpddsknshlkelegaeerltlhkvdlldle<br>svkavingcdgiihtaspvtdnpeemvepavngaknviiaaaeakvrrvvftssigavymdpsrnidevvdescwsn<br>leyckntknwycygkavaeqaawdeakargvdlvvvnpvlvlgpllqstmnastihilkyltgsaktyanatqayvhv<br>kdvalahvlvyeipsasgrylcsesslhrgelveilakyfpeypiptkcsdeknprakaytfsnkrlkdlgleftpvhqcly<br>dtvkslqdkghlplptk
PrCCR	AFC38436.1	mtagkqteegqtvcvtgaggfiaswlvklllergytvrgtvrnpedqknahlkqlegaeerltlvkadlmdynsllnain<br>gcqgvfhvaspvtddpeemvepavngtknvldacavagvrrvvftssigavymdpsrdydalvdencwsnldyck<br>etknwycygktvaekaawerakdkgldlvvvnpcvvlgpvlqssinasiihilkyltgsaktyansvqayvhvrdvae<br>ahilvyespsasgrylcaesvlhrgdvvdllasmfpqypiptkvkedgkprvkpwkvsnqklkdlgleftpakqclyet<br>vislqekghisk
PaCCR	CAK18610.1	mtagkqtgagqtvcvtgaggfiaswlvklllergytvrgtvrnpedqknahlrqlegaeerltlvkadlmdynsllnaitg<br>cqgvfhvaspvtddpvqmvepavngtknvldacaeaavrrvvftssigavymdptrdydalvdescwsnldfckdt<br>knwycygkavaekaawdrakekgldlvvvnpcvvlgpvlqssinasilhilkyltgsaktyansvqayvhvrdvaea<br>hilvyespsasgrylcaesvlhrgdvvellekmfpqypiptkckddgkprvkpwkvsnqklkdlgleftpakqclyet<br>vislqekghi
ZmCCR1	NP001105488<br>.1	mtvvdavvsstdagapaaaatavpagngqtvcvtgaagyiaswlvklllekgytvkgtvrnpddpknahlkaldgaa<br>erlilckadlldydaicravqgcqgvfhtaspvtddpeqmvepavrgteyvinaaaeagtvrrvvftssigavtmdpkrg<br>pdvvvdescwsdlefcektrnwycygkavaehaawetarrrgvdlvvvnpvlvvgpllqatvnasiahilkyldgsar<br>tfanavqayvdvrdvadahlrvfesprasgrhlcaervlhredvvrilaklfpeypvparcsdevnprkqpykfsnqklr<br>dlglqfrpvsqslydtvknlqekghlpvlgertttteaadkdaptaemqqggiaira
TaCCR1	ABE01883.1	mtvvaaaaaaaaqelpghgqtvcvtgaagyiaswlvklllergytvkgtvrnpddpknahlkaldgaaerlvlckadll<br>dydaicaavegchgvfhtaspvtddpeqmvepavrgteyvinaaadagtvrrvgvtssigavtmdpnrgpdvvvde<br>scwsdlefckktknwycygkavaeqaawekaaargvdlvvvnpvlvvgpllqptvnasaahilkyldgsakkyana<br>vqayvnvrdvaaahvrvfeapgasgrhlcaervlhredvvhilgklfpeypvptrcsdevnprkqpykmsnqklqdl

		glqftpvndslyetvkslqekghlpaprkdilpaeldgata
TaCCR2	AAX08107.1	mpieaadnvpaelpghgrtvcvtgaggfiaswlvkrllqkgynvrgtvrnpvdpkndhlrafdgaadrlvllradlme<br>petlveaftgcegifhaaspvtddpekmiepairgtkyvitaaadmgikrvvftstigtvymnpnrdpskpvddtcwsd<br>leyckktanwycyaktvaeqdaletarqrgielivvnpvlvlgpllqptvnastehvmkyltgsaktyvnaaqayvhvk<br>dvaeahvrvyeapgahgryicaegttlhrgelcrvlcklfpeypvptkckdevnppvkgykftnqrlkdlgmefvpvl<br>qsiyetvkslqekgmlpvlppgddvrdnlheqlmmkpaqllrn
ZmCCR2	NP001105715.1	mptaeattpvppelssgqgrtvcvtgaggfiaswlvkrllekgytvrgtvrnpvdpkndhlraldgavdrlvllradlldp<br>qslaeafsgcdgvfhaaspvtddpemmiepairgtryvmaaaddagvkrvvftssigtvymnpyrdpskpvddtcw<br>sdleyckntqnwycyaktvaeqgawevarkrgldlvvvnpvlvlgpllqptvnastdhvmkyltgsattyvnaaqay<br>vhvrdvaeahvrvyeaphahgryicaestlhrgdlcrvlaklfpeypvptkckdqvnppvvgykftnqrlkdlgmdfv<br>pvlqclyetvtslqekgmlpvlptkqdhdqlgks
StCCR	NP001274901.1	mpsesgkvvcvtgaggfiaswlvklllekgytvrgtvrnpddpknghlkelegakerlillradlldyqslreaiygcdg<br>vfhtaspvtddpeqmvepavigtknvitaaaeakvgrvvftssigtvymdpnrapdkvvdetcwsdlgfckntknw<br>ycygktvaektawdearekgvdlvvinpvlvlgpllqptvnasvlhilkyltgsaktyansiqayvhvkdvalahillye<br>apsasgryicaesvlhrgdvveilakffpeypiptkcsdetrprakpykftnqklkdlglgftpvkqclyetvkslqekghl<br>piptqndepikihs
OsCCR	CAD21520.1	mssnfeannnngekqlvcvtgaggfigswvvkellirgyhvrgtardpadsknahllelegadqrlslcradvldaaslr<br>aafsgchgvfhvaspvsndpdlvpvavegtrnvinaaadmgvrrvvftssygavhmnpsrspdavldetcwsdyef<br>crqtdnlyccakmmaemtateeaakrglelavvvpsmtmgpmlqqtlnfssnhvarylmgtkksypnavaayvdv<br>rdvarahvlvyerpdargrylcigtvlhraellrmlrdlfpqypatakceddgkpmakpykfsnqrlkdlgleftplrksl<br>heavlcmqqkshlpliypvpkrayl
TpCCR	PNY09320.1	mstydnislvsgtdqtvcvtgaggfiaswlvklllekgytvkgtvrnpedpknshlkelegarerltlhkvdlldlksiqsv<br>ihgchgvfhtaspvtdnpeemlepavygtknviisaaeakvrrivftssigtvymnpntsrdvvvdesywsdleyckn<br>tknwycygktvaeqsawdiakenqvdlvvvnpvlvlgpllqstvnastmhilkylngsaktyvnatqsyvsvkdval<br>ahvlvyetnsasgryicsdaslhrgevveilakyfpeyplptk
PdCCR	AGS10092.1	mtvvdavssaaavaqpagngqtvcvtgaagyiaswlvklllekgytvkgtvrnpddpknahlralegaaerlilckadl<br>ldydaicravqgcqgvfhtaspvtddpeqmvepavrgteyvisaaaeagtvrrvvftssigavtmdpnrgpdvvvdes<br>cwsdlefckktrnwycygkavaeqaawdaarhrgvdlvvvnpvlvvgpllqptvnasighvlkyldgsartfanavq<br>ayvdvrdvadahvrvfespraagrhlcaervlhredvvdlaklfpeypvptrcsdevnprkqpykfsnqklrdlglefrs<br>vsqslydtvkclqekghlpvpvpdeqtateakkktvapaaelqqggiaira
HvCCR	AAN71760.1	mtvvdaaaavaqelpghgqtvcvtgaagyiaswlvklllergytvkgtvrnpddpknahlkaldgaaerlvlckadlld<br>ydaicaavegchgvfhtaspvtddpeqmvepavrgteyvidaaadagtvrrvvftssigavtmdpnrgpdvvvdesc<br>wsdlefckktknwycygkavaeqaawekarargvdlvvvnpvlvvgpllqptvnasaahilkyldgsarkyanavq<br>ayvdvrdvagahlrvfeapqasgrylcaervlhrqdvvhilaklfpeypvptrcsdevnprkqpykmsnqklqdlglk<br>ftpvndslyetvkslqekghlpvprkdilapqldgata
MtCCR1	XP003604238.1	mpaydntssvsggdqtvcvtgaggfiaswlvklllergytvrgtvrnpedpknghlkelegarerltlhkvdlldlqsiqs<br>vvhgchgvfhtaspvtdnpdemlepavngtknviiasaeakvrrvvftssigtvymdpntsrdvvvdesywsdlehc<br>kntknwycygktvaeqsawdiakenqvdlvvvnpvvvlgpllqptinastihilkylngaaktyvnatqsyvhvkdv<br>alahllvyetnsasgryiccetalhrgevveilakyfpeyplptkcsdeknprvkpykfsnqklkdlgleftpvkqclydt<br>vrslqekghlpippmqedsa
MtCCR-like SNL6	MTR_5g029990	mapsfditshtvcvmdasgqlgfslvqrllqrgytvhasiqqygenpfngisadsdklkifrsdpfdyhsiidalrgcsglf<br>ysfeppldqpnydeymadvevraahnvleacaqtetidkvvftssatavvwredrktiepdldeihwsdvnfcrkfkl<br>whgmsktlaektafalamdrgvnmvsinagllmthdlsikhpylrgaaemyedgvfvtvdlgflvdthicvyedvss<br>ygrylcfnhiintqddavqlahkltptassslpqsddygksyieqkisnkklnklmvdfea
MtCCR-like SNL6 2	MTR_6g406250	mgvvrtcaikrseldifhrnlvaaagihrrkddeglrknklfsslddedvgktlvcvtsgvsylglalvnhllvlgysvriiv<br>dnpgdreklreirheemrnnleiiiaklndvdslvkafegcrgifhtsaftdpaglsgytksmaeievnvtenvmeacart<br>psikrcvftsslaaciwqdnvnsdlttpiinhgswsseslcidkklwyalgkmraekaawrianerglklttícpalitgpe<br>fchrnptatiaylkgaqkmysngllatvdvkkvaeahacvfkemnenaygryicfdnvidaqseaeklakdigmpke<br>kicgdasnislqryelsnnklcrlmsrpircssen
AtCCR-like	AT4g30470	mdhdkstscccvldastyvgfwilkkllsrgysvhaairrngeseieemiremetteerlvvydvdvldyqsilvslktc<br>nvvfccldspegydekevdlevrgainvveacgrtesiekivfsssltasiwrdnigtqkdvdekcwsdqdfcrskklw<br>halakmlsekaawalamdrrlnmvsinpglvvgpsvaqhnarptmsylkgaaqmyengvlayvdvkfladvhira<br>fedvsacgryfcfnqivnteeealklveslsplipmppryenemhgsevyeerlrnnklsklveagsac

Table S4. Primer sequences for RT-qPCR.

Primer name	sequences	Tm (°C)	Product length
Group Ia	F: TTATGTGCTGAAAGTTCACTTCAC	57.94	159 bp
	R: CTCCAATCCCAAATCCTTCAGT	58.03	
Group Ib	F: GAGGCTAACTTTGCATAAGGTTGA	59.30	161 bp
	R: ACGCTATAATCACATTCTTTGT	53.92	
Group II	F: ACAGCGTTTGCCTTAGCAATGGAC	64.40	158 bp
	R: AAAAATCCTAAATCAACGGTCA	54.28	
Group III	F: TTGTATTTGGCAAGACAATGTGAA	57.85	213 bp
	R: TGACAAAATTCAGGACCTGTGATA	58.12	
Group IV	F: AGAAGCAGCAAAACGCGTGGGT	66.01	142 bp
	R: CCCCCTCAACTTACAGTACTCAAC	60.56	
MsCCR-like17	F: CTGGACTTAACGTGAATAAG	51.38	165 bp
	R: GAAATTGAAGAGTATATATCCCA	51.24	
Actin2	F: AAAAGGATGCCTATGTTGGTG	56.48	186 bp
	R: TAAGTGGAGCCTCAGTTAGAAGTA	58.42	
